# Antihypertensive Drugs and Dental Caries Risk: A Drug–Target Mendelian Randomization Analysis

**DOI:** 10.1155/ijog/1612322

**Published:** 2025-10-07

**Authors:** Wenbin Shi, Shuhua Liu, Xiuxia Wu, Wangsong Cheng, Xiqun Jia, Ziyang Hu

**Affiliations:** ^1^ Department of Neonatal, Shenzhen Longhua District Central Hospital, Shenzhen, China, glyy.org; ^2^ Department of Stomatology, Shenzhen Longhua District Central Hospital, Shenzhen, China, glyy.org; ^3^ Department of Dentomaxillofacial Radiology, Nanjing Stomatological Hospital, Medical School of Nanjing University, Nanjing, China, nju.edu.cn

**Keywords:** antihypertensive agents, genetic predisposition to disease, genome-wide association study, Mendelian randomization analysis

## Abstract

**Introduction and Objectives:**

To address the clinical uncertainty surrounding the effect of antihypertensive drugs on dental caries, this study was aimed at investigating the causal relationships between antihypertensive medication use and the risk of dental caries, utilizing a drug–target Mendelian randomization (MR) approach.

**Methods:**

Nine antihypertensive drug classes′ influences on dental caries risk in the UK Biobank and FinnGen populations were assessed using drug–target MR. This genetic method utilizes randomly allocated gene variants as proxies for drug exposure, minimizing the confounding biases inherent in observational studies and allowing for more robust causal inference. Genetic variants associated with systolic blood pressure near the drug target genes were used to proxy for medication effects.

**Results:**

In the FinnGen cohort, genetic analysis linked calcium channel blockers to a 3.3% reduction in dental caries risk (OR: 0.967, 95% CI: 0.949–0.985) and loop diuretics to a 6.9% reduction (OR: 0.931, 95% CI: 0.897–0.966). Conversely, aldosterone antagonists were suggestively associated with an 8.2% increased risk (OR: 1.082, 95% CI: 1.017–1.150). Notably, the protective trend for calcium channel blockers and loop diuretics was also observed in the UK Biobank. These findings, validated by eQTLs, highlight the impact of antihypertensive drugs on dental health.

**Conclusion:**

The study suggests that calcium channel blockers and diuretics could potentially reduce the risk of dental caries. Additional research is needed to assess the feasibility of repurposing antihypertensive medications for the prevention of dental caries.

## 1. Introduction

Dental caries, widely recognized as tooth decay or cavities, represents a significant global health issue, affecting diverse age demographics [1]. This condition is characterized by the demineralization of tooth enamel, a direct consequence of acidic by‐products emanating from the bacterial fermentation of sugars within the oral cavity [2]. The advancement of dental caries can culminate in considerable discomfort, pain, and potential tooth loss, adversely affecting not just oral health but also the overall quality of life [3]. Consequently, the effective management and prevention of dental caries are paramount in global public health initiatives.

Hypertension, commonly referred to as high blood pressure, presents a formidable challenge to health systems worldwide [4]. It stands as a principal risk factor for cardiovascular diseases, including stroke, heart attack, and heart failure, contributing significantly to the global disease burden and mortality rates [5–7]. The management of hypertension typically encompasses lifestyle modifications and pharmacotherapy, with antihypertensive medications being crucial in controlling blood pressure levels and reducing the risk of cardiovascular incidents.

Recent investigations have begun to explore the intricate links between oral and systemic health. A consensus report affirmed a strong association between periodontitis and cardiovascular diseases, although a definitive causal pathway has not been established [8]. The relationship between hypertension and dental caries, however, remains ambiguous. While some epidemiological studies suggest a positive correlation [9, 10], others have failed to observe a significant association [11, 12]. Such conflicting results may stem from methodological limitations inherent to observational studies. For instance, it is difficult to fully account for residual confounding from shared lifestyle and socioeconomic factors. Furthermore, these studies are susceptible to reverse causation, where chronic inflammation from poor oral health could plausibly contribute to hypertension. While a direct causal link is debated, the influence of antihypertensive medications on dental caries is biologically plausible through several mechanisms. Many of these drugs, particularly diuretics, are known to induce xerostomia (dry mouth) [13], a significant risk factor that impairs saliva′s natural cleansing and buffering capacity [14]. Furthermore, studies have shown that these medications may alter salivary composition, including its pH and electrolyte levels, which are critical for enamel remineralization [15].

Therefore, elucidating the potential causal influence of antihypertensive medications on dental caries carries significant implications for clinical practice and public health policy. A confirmed causal link would necessitate the development of integrated care models that manage hypertension while mitigating adverse oral health outcomes. To date, the impact of antihypertensive drugs on caries risk has been minimally explored, and traditional pharmacoepidemiological studies are often hampered by inherent biases that can compromise their validity.

To overcome these limitations, we employ Mendelian randomization (MR), an analytical method that uses genetic variants as instrumental variables to assess the causal relationship between a modifiable exposure and a health outcome [16]. By leveraging the random allocation of genes from parents to offspring, MR uses genetic variants as instrumental variables to assess causality. This approach is inherently more robust against the residual confounding that plagues observational research and is not susceptible to reverse causation, offering more reliable causal inference than is often feasible with long‐term randomized controlled trials (RCTs) [17]. MR diminishes the bias from confounding variables typical in observational research and offers practical advantages over RCTs. Drug–target MR, a novel iteration of this approach, pre‐emptively evaluates the outcomes and side effects of therapeutic interventions by utilizing genetic variants as proxies for the effects of drug targets, facilitating the exploration of protein functionalities and the implications of modifying these targets. For instance, this framework has been successfully used to validate the therapeutic effects of lipid‐lowering drugs (targeting HMGCR) on coronary artery disease (CAD) [18] and to explore off‐target effects of antihypertensive drug classes on cancer risk [19, 20], demonstrating its power in pharmacological research.

The novelty of our study lies in its specific application of the drug–target MR framework to systematically investigate the causal effects of nine distinct classes of antihypertensive drugs on dental caries risk. Drug–target MR enables a more precise exploration of the pharmacological pathways by using genetic variants within or near drug target genes as proxies for medication effects. This provides a unique opportunity to dissect how these widely used medications influence oral health, an endeavor nearly impossible to achieve conclusively with conventional methods. Accordingly, this study is aimed at using a drug–target MR framework to systematically investigate two primary questions: (1) whether genetically predicted hypertension has a causal effect on dental caries risk and (2) whether genetic proxies for nine major classes of antihypertensive medications causally modify this risk.

## 2. Materials and Methods

### 2.1. Study Design

**Figure 1:**
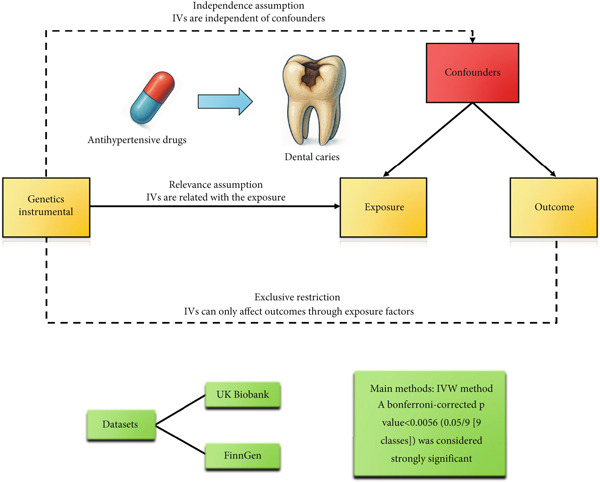
Study design of the MR study of the associations between genetically proxied antihypertensive drugs and the risk of dental caries.

The drug–target MR study was performed to assess the causal effects of various antihypertensive medication classes on dental caries risk. This approach was chosen to overcome the limitations of other methods; it mitigates the residual confounding and reverse causation inherent in observational studies and, unlike standard MR of blood pressure, can isolate the effects of specific pharmacological mechanisms [21]. By using genetic variants as proxies for drug targets, this framework simulates each medication′s specific intervention, directly testing its biological plausibility. The overall study workflow is depicted in Figure 1.

First, we identified nine major classes of antihypertensive drugs from the British National Formulary and their corresponding protein targets using the DrugBank database. Next, we selected genetic variants associated with systolic blood pressure (SBP) located in or near the genes encoding these protein targets to serve as genetic instruments. These instruments were sourced from a large‐scale genome‐wide association study (GWAS) meta‐analysis of individuals of European ancestry.

To validate the genetic instruments, a positive control analysis was performed using CAD as the outcome. This step ensures the genetic proxies replicate the known protective cardiovascular effect of these drugs, confirming they accurately simulate the intended pharmacological action [21]. This analysis was for instrument validation only and does not imply any biological link between CAD and dental caries. For a comprehensive assessment, all drug classes were retained for the primary analysis, regardless of whether their genetic proxies showed a significant protective effect on CAD (*p* < 0.05).

Finally, using the validated instruments, we conducted a two‐sample MR analysis to estimate the causal effect of each antihypertensive drug class on the risk of dental caries in the UK Biobank and FinnGen cohorts. A secondary analysis using cis‐expression quantitative trait loci (cis‐eQTLs) was also performed to corroborate the findings.

### 2.2. Genetic Instrument Selection for Antihypertensive Drug Classes

We identified nine classes of antihypertensive drugs: adrenergic neuron blockers, alpha‐adrenoceptor blockers, ACE inhibitors (ACEi), angiotensin II receptor blockers (ARBs), beta‐blockers (BBs), calcium channel blockers (CCBs), loop diuretics, potassium‐sparing diuretics and aldosterone antagonists, thiazide diuretics, and related compounds [22]. The specific genes encoding the protein targets for each drug class were identified from the DrugBank database and are listed in Supporting Information 1: Table S1.

To serve as genetic proxies for the effects of these antihypertensive drugs, we selected single nucleotide polymorphisms (SNPs) associated with SBP from a meta‐analysis of the International Consortium of Blood Pressure (ICBP) and UK Biobank, which included 757,601 individuals of European descent. We selected independent, genome‐wide significant SNPs (*p* < 5 × 10^−8^) located within a ±100 kb radius of the target genes for each drug class, ensuring these SNPs had low linkage disequilibrium (*r* ^2^ < 0.1 within 100 kb) with each other [19]. This window was chosen based on common practice in drug–target MR studies, as it is wide enough to capture cis‐acting regulatory elements that influence gene function without extending into regions likely to contain unrelated genes, thus minimizing potential pleiotropy [19, 23]. The strength of each genetic instrument was evaluated using the *F*‐statistic, and instruments with an *F*‐statistic below 10 were excluded to mitigate weak instrument bias.

When a selected SNP was unavailable in an outcome GWAS, we used LDlink to identify a suitable proxy SNP in high LD (*r* ^2^ > 0.8). If no appropriate proxy was found, the SNP was removed from the analysis (10 SNPs replaced with proxies, three SNPs excluded). Also, we harmonized the exposure and outcome datasets to ensure that the effect alleles were consistent. Palindromic SNPs and those with ambiguous alleles (minor allele frequency > 0.42) were excluded.

### 2.3. Outcome Data

In this study, CAD served as the control outcome, reflecting the pivotal role of antihypertensive medications in decreasing CAD morbidity. For Europeans, summary‐level CAD data were derived from a combined analysis of GWAS conducted by the CARDIoGRAMplusC4D consortium and the UK Biobank, encompassing 122,733 CAD cases and 424,528 controls [24].

The primary outcome was dental caries. We obtained summary‐level data from two large European‐ancestry cohorts.

UK Biobank: Summary‐level dental caries data were obtained from a GWAS involving 361,194 individuals of European descent from the UK Biobank, which included 2110 caries cases and 359,084 controls. Dental caries was classified according to the International Classification of Diseases, Ninth Revision (ICD‐9) and Tenth Revision (ICD‐10), with codes 521 and K02.9, respectively, for each revision [25]. The GWAS utilized the BOLT‐LMM method for analysis, and the resulting association statistics, initially presented on a linear scale, were converted into log odds ratios (ORs) using a standardized transformation process.

FinnGen cohort: Summary‐level dental caries data came from a GWAS of 199,565 individuals, which recorded 4170 caries cases and 195,395 controls. The FinnGen consortium functions as a multi‐institutional, hospital‐based registry that compiled DNA, serum, and clinical data from roughly 200,000 patients across 66 hospitals during the period of 2003–2008. In this hospital‐based cohort, cases were defined by physician diagnoses recorded in national health registries [26].

### 2.4. Statistical Analysis

#### 2.4.1. Validation of Genetic Instruments

All analyses were conducted assuming sufficient statistical power due to the large sample sizes of the underlying GWAS. However, we acknowledge that for drug classes with fewer or weaker genetic instruments, null findings should be interpreted with caution, as study power may be insufficient to detect very small effects (Supporting Information 2: Table S2).

First, we performed an MR analysis with each antihypertensive drug class as the exposure and CAD as the outcome to validate our genetic instruments. The inverse‐variance weighted (IVW) method was used to estimate the causal effect. Any drug class that did not show a nominally significant protective effect on CAD risk (*p* < 0.05) was excluded from further analysis, as this would suggest that the genetic instruments were not validly proxying the drug′s antihypertensive action.

#### 2.4.2. Primary MR Analysis

Next, we used the validated genetic instruments to estimate the causal effect of each antihypertensive drug class on the risk of dental caries in both the UK Biobank and FinnGen cohorts. The IVW method was again employed for the main analysis. In the presence of heterogeneity, a multiplicative random‐effects IVW model was used; otherwise, a fixed‐effect model was applied. Heterogeneity was assessed using Cochran′s *Q* test and the *I* ^2^ statistic [27]. The results are presented as ORs with 95% confidence intervals (CIs) for dental caries per 1 mmHg reduction in SBP genetically predicted by the drug target. To account for multiple testing, we applied a Bonferroni correction, with a *p* value of 0.0056 (0.05/9) considered statistically significant and a *p* value between 0.0056 and 0.05 considered suggestive of an association.

#### 2.4.3. Sensitivity Analyses

To assess the robustness of our findings, we conducted several sensitivity analyses. We used MR‐Egger regression to detect and adjust for horizontal pleiotropy, where the intercept term serves as an indicator of directional pleiotropy. The MR‐PRESSO (Pleiotropy Residual Sum and Outlier) test was also used to identify and correct for pleiotropic outliers [28, 29]. Also, a leave‐one‐out analysis was performed to determine if the causal estimates were driven by any single SNP.

#### 2.4.4. Secondary Analysis Using cis‐eQTLs

To further strengthen our findings, we conducted a secondary MR analysis using cis‐eQTLs as genetic instruments to corroborate the primary findings and explore gene expression as a potential mechanism. cis‐eQTLs were prioritized because they directly regulate the drug target′s gene expression, offering a more specific proxy for the medication′s effect. This exploratory analysis was limited to cis‐eQTLs and did not test other genomic annotations. These eQTLs were selected independently from the primary SBP‐associated instruments to serve as a distinct set of genetic proxies operating through gene expression. We sourced cis‐eQTLs from the Genotype‐Tissue Expression (GTEx) portal [22], prioritizing those from orally relevant tissues where available to enhance biological plausibility. We first confirmed the association of these eQTLs with SBP and then estimated their causal effect on dental caries. This approach allowed us to triangulate our findings by using a different type of genetic instrument that proxies the drug′s mechanism of action through gene expression.

All statistical analyses were performed in R (Version 4.2.2) using the “TwoSampleMR” and “MRPRESSO” packages.

## 3. Results

### 3.1. Genetic Instrument Selection

We selected between 7 and 155 SNPs associated with SBP to serve as genetic instruments for nine classes of antihypertensive drugs. All selected SNPs exhibited *F*‐statistics well above the threshold of 10, indicating strong instrument strength and a low risk of weak instrument bias. The distribution of *F*‐statistics for the instrument sets for each drug class ranged from a minimum of 29.7 to a maximum of 352.5, with a median of 57.8 (Supporting Information 3: Table S3).

**Figure 2:**
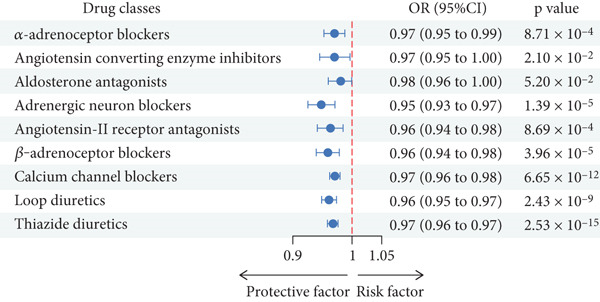
Associations between genetic proxies for nine classes of antihypertensive drugs and the risk of CAD.

In our positive control analysis, the genetic proxies for eight of the nine drug classes demonstrated a statistically significant protective association with the risk of CAD, validating their efficacy as instruments that mimic the drugs′ antihypertensive effects. The genetic proxy for aldosterone antagonists did not show a significant association with CAD risk; however, this class was retained for the primary analysis to ensure a comprehensive assessment of all major antihypertensive categories (Figure 2).

### 3.2. Causal Effect of Genetically Proxied Antihypertensive Drugs and Dental Caries

**Figure 3:**
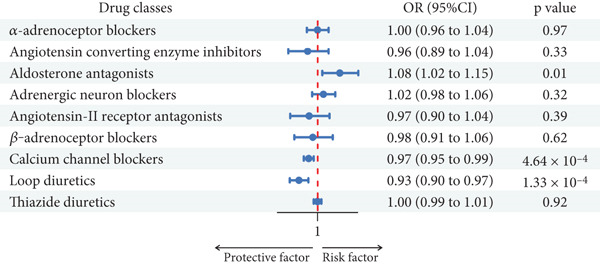
Associations between genetic proxies for nine classes of antihypertensive drugs and the risk of dental caries in FinnGen.

**Figure 4:**
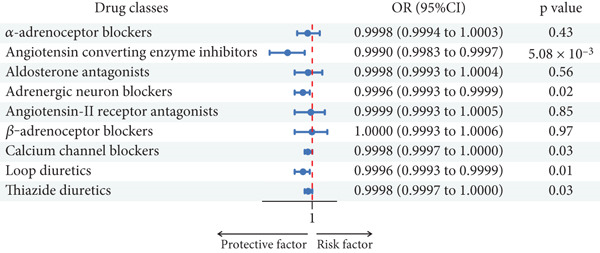
Associations between genetic proxies for nine classes of antihypertensive drugs and the risk of dental caries in UK Biobank.

Using the IVW method, we discovered robust evidence linking genetically proxied angiotensin converting enzyme inhibitors with a reduced risk of dental caries in UKBB (OR: 0.9990; 95% CI: 0.9983–0.9997 per 1 mmHg reduction in SBP; *p* = 5.08 × 10^−3^). Genetically proxied CCBs (OR: 0.9668; 95% CI: 0.9488–0.9853 per 1 mmHg reduction in SBP; *p* = 4.64 × 10^−4^) and loop diuretics showed a reduced risk of dental caries in FinnGen (OR: 0.9308; 95% CI: 0.8971–0.9657 per 1 mmHg reduction in SBP; *p* = 1.33 × 10^−4^) but showed no association in UK Biobank (Table 1, Figures 3 and 4).

**Table 1:** Primary results for MR of genetic proxies for nine antihypertensive drugs with dental caries.

	**Drugs**	**Beta**	**SE**	**p** **value**	**OR**	**95% CI**
UK Biobank	Alpha‐adrenoceptor blockers	−0.0002	0.0002	0.4353	0.9998	0.9994–1.0003
	Angiotensin‐converting enzyme inhibitors	−0.0010	0.0004	**5.08e**−**03**	0.9990	0.9983–0.9997
	Aldosterone antagonists	−0.0002	0.0003	0.5690	0.9998	0.9993–1.0004
	Adrenergic neuron blockers	−0.0004	0.0002	**0.0192**	0.9996	0.9993–0.9999
	Angiotensin II receptor antagonists	−0.0001	0.0003	0.8544	0.9999	0.9993–1.0005
	Beta‐adrenoceptor blockers	0.0000	0.0003	0.9726	1.0000	0.9993–1.0006
	Calcium channel blockers	−0.0002	0.0001	**0.0296**	0.9998	0.9997–1.0000
	Loop diuretics	−0.0004	0.0001	**0.0117**	0.9996	0.9993–0.9999
	Thiazide diuretics	−0.0002	0.0001	**0.0262**	0.9998	0.9997–1.0000

FinnGen	Alpha‐adrenoceptor blockers	−0.0006	0.0205	0.9778	0.9994	0.9602–1.0403
	Angiotensin converting enzyme inhibitors	−0.0378	0.0394	0.3374	0.9629	0.8914–1.0402
	Aldosterone antagonists	0.0783	0.0313	**0.0123**	1.0815	1.0171–1.1499
	Adrenergic neuron blockers	0.0210	0.0213	0.3244	1.0212	0.9795–1.0647
	Angiotensin II receptor antagonists	−0.0321	0.0382	0.3999	0.9684	0.8986–1.0436
	Beta‐adrenoceptor blockers	−0.0180	0.0368	0.6240	0.9821	0.9138–1.0556
	Calcium channel blockers	−0.0337	0.0096	**4.64e**−**04**	0.9668	0.9488–0.9853
	Loop diuretics	−0.0717	0.0188	**1.33e**−**04**	0.9308	0.8971–0.9657
	Thiazide diuretics	−0.0007	0.0073	9.27e−01	0.9993	0.9852–1.0137

*Note:* Bold entries indicate *p* < 0.05.

Furthermore, in the FinnGen cohort, suggestive evidence indicated that genetically proxied aldosterone antagonists might increase the risk of dental caries (OR: 1.0815; 95% CI: 1.0171–1.1499 per 1 mmHg reduction in SBP; *p* = 0.0123). For UK Biobank, moderate evidence supported that genetically proxied adrenergic neuron blockers (OR: 0.9996; 95% CI: 0.9993–0.9999 per 1 mmHg reduction in SBP; *p* = 0.0192), CCBs (OR: 0.9998; 95% CI: 0.9997–1.000 per 1 mmHg reduction in SBP; *p* = 0.0296), loop diuretics (OR: 0.9996; 95% CI: 0.9993–0.9999 per 1 mmHg reduction in SBP; *p* = 0.0117), and thiazide‐related diuretics (OR: 0.9998; 95% CI: 0.9997–1.0000 per 1 mmHg reduction in SBP; *p* = 0.0262) were linked to a lower risk of dental caries (Table 1, Figures 3 and 4).

To assess the robustness of these findings, we conducted a series of sensitivity analyses (Table 2 and Supporting Information 4: Table S4). The primary causal estimates were largely consistent across different MR methods, including the weighted median and MR‐PRESSO approaches.

**Table 2:** Heterogeneity and pleiotropy results.

	**Drugs**	**Heterogeneity**		**Pleiotropy**		
		**Q**	**p** **value**	**Egger_intercept**	**SE**	**p** **value**
UK Biobank	Alpha‐adrenoceptor blockers	25.679	0.099	−5.06e−05	1.61e−04	0.757
	Angiotensin‐converting enzyme inhibitors	3.987	0.678	1.93e−04	2.63e−04	0.497
	Aldosterone antagonists	9.810	0.366	−3.99e−04	2.21e−04	0.109
	Adrenergic neuron blockers	20.898	0.645	−2.87e−04	1.45e−04	0.059
	Angiotensin II receptor antagonists	6.386	0.604	4.95e−06	2.56e−04	0.985
	Beta‐adrenoceptor blockers	11.043	0.199	7.64e−05	2.09e−04	0.726
	Calcium channel blockers	102.000	0.509	3.51e−05	6.58e−05	0.595
	Loop diuretics	24.261	0.668	1.83e−04	1.42e−04	0.207
	Thiazide diuretics	208.681	0.002	5.87e−05	5.82e−05	0.315

FinnGen	Alpha‐adrenoceptor blockers	22.711	0.537	6.00e−03	1.33e−02	0.657
	Angiotensin‐converting enzyme inhibitors	5.885	0.436	3.32e−03	3.19e−02	0.921
	Aldosterone antagonists	4.529	0.873	2.15e−02	2.42e−02	0.401
	Adrenergic neuron blockers	13.219	0.947	−1.32e−02	2.01e−02	0.519
	Angiotensin II receptor antagonists	3.798	0.704	3.16e−02	3.44e−02	0.400
	Beta‐adrenoceptor blockers	6.165	0.521	−2.44e−02	2.37e−02	0.344
	Calcium channel blockers	85.313	0.883	7.14e−03	7.83e−03	0.364
	Loop diuretics	23.951	0.522	2.73e−02	1.81e−02	0.145
	Thiazide diuretics	115.840	0.976	7.14e−04	5.82e−03	0.903

The MR‐Egger intercepts and MR‐PRESSO global tests showed no evidence of significant directional horizontal pleiotropy for any of the tested associations. Significant heterogeneity was detected among the instruments for thiazide diuretics in the UK Biobank (Cochran′s *Q* value = 208.681, *p* = 0.002), and a multiplicative random‐effects IVW model was therefore used for this analysis as prespecified. The MR‐PRESSO outlier test confirmed the IVW estimates, providing robust results. For example, the protective effect of CCBs on dental caries risk in the FinnGen cohort remained highly significant in the MR‐PRESSO analysis (OR: 0.9709; 95% CI: 0.9503–0.9837; *p* = 2.23 × 10^−4^) reinforcing the validity of our primary findings.

### 3.3. Causal Effect of Expressions of Target Genes of Antihypertensive Drugs and Dental Caries

Upon refining the cis‐eQTLs from previous research by clumping (*r* ^2^ < 0.1), we identified eQTLs for the nine classes of antihypertensive drugs, detailed in Supporting Information 5: Table S5. Consistently, reduced expression levels of the target genes for these antihypertensive drug classes correlated with lower SBP across the board (all *p* < 0.001).

Furthermore, our analysis revealed that decreased expression of target genes for CCBs (beta: −1.40 [−2.23 to −0.57] per 1 mmHg decrease in SBP; *p* = 9.64 × 10^−4^) and loop diuretics (beta: −2.98 [−5.69 to −0.27] per 1 mmHg decrease in SBP; *p* = 0.03) was linked to reduced risks of dental caries in FinnGen cohort (Supporting Information 6: Figure S1). Correspondingly, expression levels of target genes for loop diuretics (beta: −0.03 [−0.06 to −0.01] per 1 mmHg decrease in SBP; *p* = 0.02) was conversely associated with risk of dental caries in UK Biobank cohort (Supporting Information 7: Figure S2).

## 4. Discussion

This MR study presents robust evidence for a protective association between genetically proxied CCBs, as well as thiazide and related diuretics, and a reduced risk of dental caries. These findings are corroborated by multiple MR analyses and eQTL studies that link the expression of target genes for these antihypertensive classes to dental caries. Our data further suggest that ACEi and loop diuretics may represent viable candidates for preventative strategies against dental caries, partly owing to their established safety profiles as antihypertensive agents. Conversely, this research did not yield conclusive evidence linking genetically proxied angiotensin receptor blockers or other primary antihypertensive treatments to a decreased risk of dental caries.

Our study represents a novel investigation into the potential influence of antihypertensive medications—specifically CCBs and loop diuretics—on the risk of dental caries. This is particularly significant given the scarcity of observational studies exploring this association. By addressing this knowledge gap, our investigation opens new avenues for therapeutic interventions. Elucidating the mechanisms that underpin these relationships is crucial for developing integrated healthcare strategies that encompass both cardiovascular and dental well‐being.

Calcium plays a pivotal role in the formation and maintenance of dental enamel, the outermost layer that protects teeth against caries [22, 30]. By modulating the influx of calcium ions into cells, CCBs may influence the enamel remineralization process [31, 32]. Remineralization is a critical defense mechanism against caries, repairing enamel by replenishing minerals lost during acid attacks from plaque bacteria [33, 34]. Consequently, the observed reduction in caries risk with CCBs may be attributed to an enhancement of enamel′s resistance to demineralization [35, 36], potentially through an indirect mechanism involving calcium homeostasis in saliva or gingival crevicular fluid. However, the proposed biological mechanisms for these findings are speculative and require direct experimental validation. For instance, the hypothesis that CCBs protect teeth by influencing enamel remineralization lacks direct in vitro evidence. Alternative explanations, such as off‐target or systemic drug effects that indirectly impact the oral environment, must also be considered.

Our findings also associate loop diuretics, such as furosemide [37], with a decreased risk of dental caries. A plausible explanation involves the effect of these diuretics on salivary flow and composition [38]. While diuretics are often linked to xerostomia (dry mouth), a condition that can elevate caries risk, loop diuretics might exert a distinct effect. It is conceivable that they alter salivary composition in a manner that creates a less favorable environment for bacterial proliferation or acid production, thereby mitigating caries risk. Further investigation is required to elucidate the precise mechanism.

ACEi are known for their role in reducing blood pressure by preventing the conversion of angiotensin I to angiotensin II, a potent vasoconstrictor [39, 40]. Their potential influence on dental caries, however, may stem from their anti‐inflammatory properties. As chronic inflammation is a known risk factor for both caries and periodontal disease [41], the anti‐inflammatory action of ACEi could confer indirect protection by improving gingival health and modulating the composition of gingival crevicular fluid, which can otherwise serve as a nutrient source for cariogenic bacteria.

Conversely, our analysis indicated that aldosterone antagonists, a class of potassium‐sparing diuretics [42], were associated with a potential increase in caries risk. Spironolactone′s mechanism of action on aldosterone receptors might unfavorably alter the oral microbiome or salivary production [43] For instance, modifications to the electrolyte balance in saliva could compromise its buffering capacity, rendering the oral environment more acidic and thus more conducive to caries development [44].

However, it is crucial to consider the potential for horizontal pleiotropy to bias the results; this is another key limitation. While our sensitivity analyses did not detect directional pleiotropy, balanced pleiotropy could still influence the findings, particularly for drugs with complex mechanisms like ACEi and aldosterone antagonists. For example, genetic instruments for ACEi may affect caries risk through pathways unrelated to blood pressure, such as systemic inflammation [45], and a similar bias could apply to the results for aldosterone antagonists.

Moreover, thiazide diuretics demonstrated a potential protective effect against dental caries. Analogous to loop diuretics, thiazides may act on saliva flow and composition [46]. Their capacity to reduce plasma volume while increasing calcium reabsorption could also be a contributing factor [47]. By potentially increasing systemic calcium availability, thiazides might enrich the calcium concentration in saliva, thereby promoting enamel remineralization and reducing caries susceptibility [48, 49].

Our findings also contribute to a broader understanding of the interplay between cardiovascular medications and oral health, which extends beyond dental caries. The inflammatory pathways modulated by drugs like ACEi are also central to the pathogenesis of periodontitis. The established bidirectional link between periodontitis and cardiovascular disease, potentially mediated by systemic inflammation, highlights a shared biological axis [8]. Therefore, the effects of antihypertensive medications on oral tissues may not be isolated to a single condition but could represent a more holistic impact on the oral inflammatory state, which warrants deeper, integrated investigation in future studies.

While this study opens exciting avenues for drug repurposing, the translational hurdles between a MR finding and clinical application are substantial. Firstly, MR estimates the effect of lifelong, modest target inhibition, which may not correspond to the dose‐dependent and acute effects of pharmacological treatment. Secondly, off‐target effects, which are not captured by drug–target MR, are a major consideration for safety. Thirdly, significant ethical and practical challenges exist in prescribing cardiovascular agents to normotensive individuals for caries prevention. Therefore, before any clinical consideration, these findings must be validated through dose–response trials and pharmacokinetic studies to establish a safe and effective therapeutic window for any potential preventative use [50].

Our study has several notable strengths. Firstly, by employing genetic variants as proxies for antihypertensive drug effects, our MR approach inherently mitigates common observational biases such as reverse causation and confounding, while bypassing the significant time and cost of RCTs. Secondly, the use of GWAS data from the largest available consortia enhances the statistical power and credibility of our findings. Thirdly, the meticulous selection of genetic variants within drug target genes, coupled with a positive control analysis, validates the suitability of our genetic instruments. Lastly, a comprehensive suite of sensitivity analyses was conducted to affirm the robustness and consistency of our results [51].

Nevertheless, certain limitations must be considered when interpreting our findings. Beyond the inherent assumptions required for causal inference in any MR analysis, this study has several specific limitations. Firstly, our study did not stratify outcomes by different types of dental caries, which would be challenging due to limitations in statistical power. Secondly, the use of ICD codes and physician diagnoses to define caries cases introduces a risk of misclassification bias, though the large sample sizes help to mitigate this issue. Thirdly, the minute, statistically significant effects found in the UK Biobank likely reflect high statistical power rather than strong biological relevance, requiring cautious clinical interpretation. Fourthly, the eQTL analysis is limited by inconsistent effects between cohorts and its reliance on strong, simplifying assumptions about gene expression, warranting cautious interpretation. Fifthly, the potential for bias from balanced or unknown horizontal pleiotropy remains a limitation for thiazide diuretics, especially for drugs with complex systemic roles, warranting future exploration with subgroup analyses. Sixthly, the generalizability of these findings is limited as the analysis was restricted to European‐ancestry populations, and replication in more diverse groups is essential to confirm these results globally. Finally, while MR can estimate the lifelong effects of genetically predicted variables, the influence of other confounding factors acquired during adulthood cannot be fully excluded. To mitigate potential biases, we did exclude SNPs associated with mortality that were proxied by enrollment age.

## 5. Conclusion

In conclusion, this MR study provides hypothesis‐generating evidence that CCBs and loop diuretics may protect against dental caries. Further research, from mechanistic studies to clinical trials, is essential to validate these findings. If such research validates these initial findings, it may contribute to improved oral health outcomes on a broader scale by opening new avenues for caries prevention.

## Ethics Statement

All the data used in this research can be found in public databases. No additional ethical approval was required.

## Disclosure

The manuscript is approved by all authors for publication. All authors gave final approval and agree to be accountable for all aspects of the work.

## Conflicts of Interest

The authors declare no conflicts of interest.

## Author Contributions

Wenbin Shi and Shuhua Liu contributed to the writing of this manuscript, formal analysis and visualization, conceptualization, supervision, methodology, and review and editing of this manuscript. Xiuxia Wua and Wangsong Cheng contributed to resources and validation. Xiqun Jia and Ziyang Hu contributed to conceptualization, methodology, project administration, funding, and review and editing. Wenbin Shi and Shuhua Liu are co‐first authors.

## Funding

No funding was received for this manuscript.

## General Statement

*Patient and Public Involvement.* Patients or the public were not involved in the design, or conduct, or reporting, or dissemination plans of our research.

## Supporting Information

Additional supporting information can be found online in the Supporting Information section.

## Supporting information


**Supporting Information 1** Table S1. Drug classes, substances, and targets with their DrugBank ID.

**Supporting Information 2** Table S2. Summary of MR methods and sensitivity tests.

**Supporting Information 3** Table S3. Genetic proxies for nine antihypertensive drug classes in Europeans.

**Supporting Information 4** Table S4. MR of genetic proxies for nine antihypertensive drugs with dental caries.

**Supporting Information 5** Table S5. Genetic proxies for nine antihypertensive drug classes from expression quantitative trait loci in GTEx.

**Supporting Information 6** Figure S1. Association between target gene expression for antihypertensive drug classes and the risk of dental caries in the FinnGen cohort.

**Supporting Information 7** Figure S2. Association between target gene expression for antihypertensive drug classes and the risk of dental caries in the UK Biobank cohort.

## Data Availability

The datasets analyzed during the current study are publicly available. Summary statistics for dental caries were obtained from the IEU OpenGWAS project (https://opengwas.io/), which includes data from UK Biobank (ID: ukb‐d‐K02) and FinnGen (ID: finn‐b‐K11_CARIES). Information on drug targets was sourced from the DrugBank database (https://go.drugbank.com/). The cis‐eQTL data were retrieved from the GTEx Portal (https://gtexportal.org/). All original data sources are cited within the manuscript. Any additional data or results from this research can be obtained by contacting the corresponding authors.
