# Supplementary figures and images for: Antihypertensive Drugs and Dental Caries Risk: A Drug–Target Mendelian Randomization Analysis
Source: Int J Genomics. 2025 Oct 7;2025:1612322. doi: 10.1155/ijog/1612322 (PMC12501493; doi:10.1155/ijog/1612322)

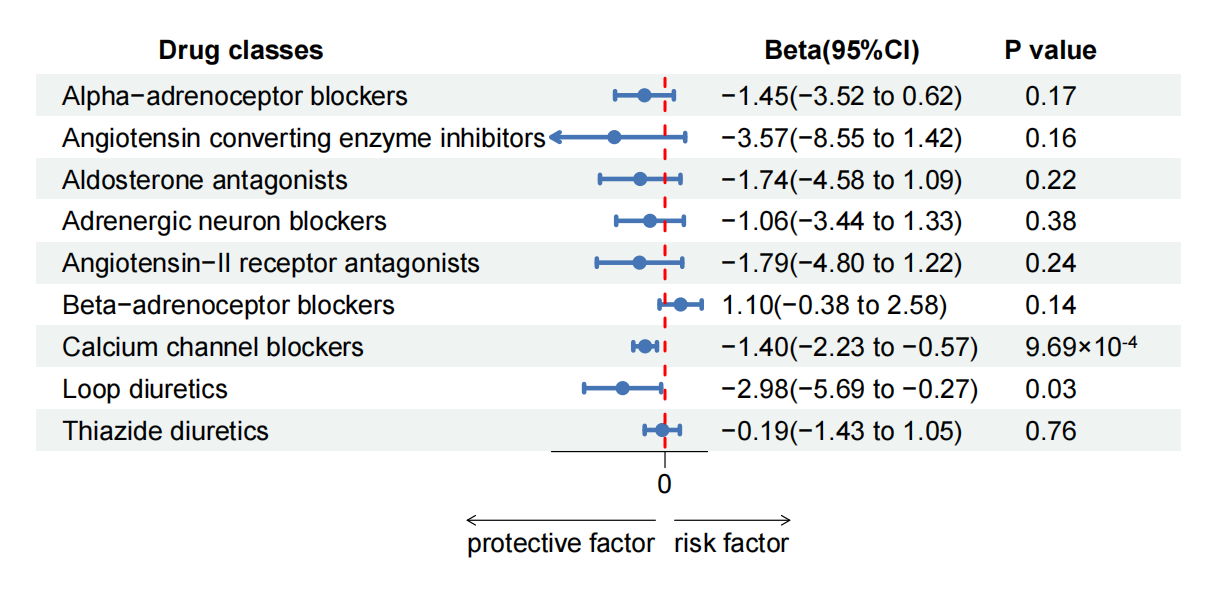

Supplement: Supplementary file 6 — Supporting Information 6 Figure S1. Association between target gene expression for antihypertensive drug classes and the risk of dental caries in the FinnGen cohort. [file IJOG-2025-1612322-s002.png]

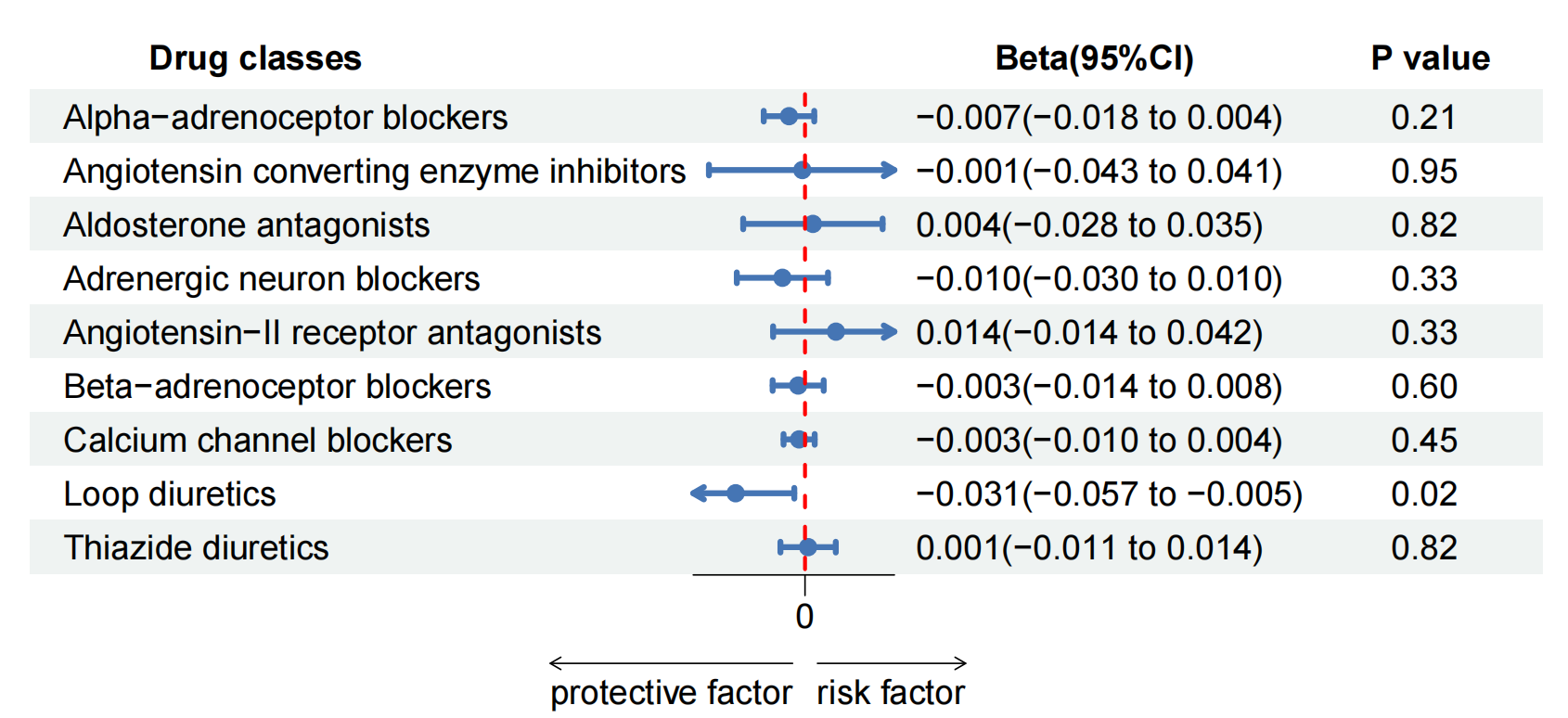

Supplement: Supplementary file 7 — Supporting Information 7 Figure S2. Association between target gene expression for antihypertensive drug classes and the risk of dental caries in the UK Biobank cohort. [file IJOG-2025-1612322-s006.png]
